# Supplementary material for: Differential Regulation of POC5 by ERα in Human Normal and Scoliotic Cells
Source: Genes (Basel). 2023 May 19;14(5):1111. doi: 10.3390/genes14051111 (PMC10218682; doi:10.3390/genes14051111)
Supplement: Supplementary file 1 [file genes-14-01111-s001.zip › genes-2395069-supplementary.pdf]

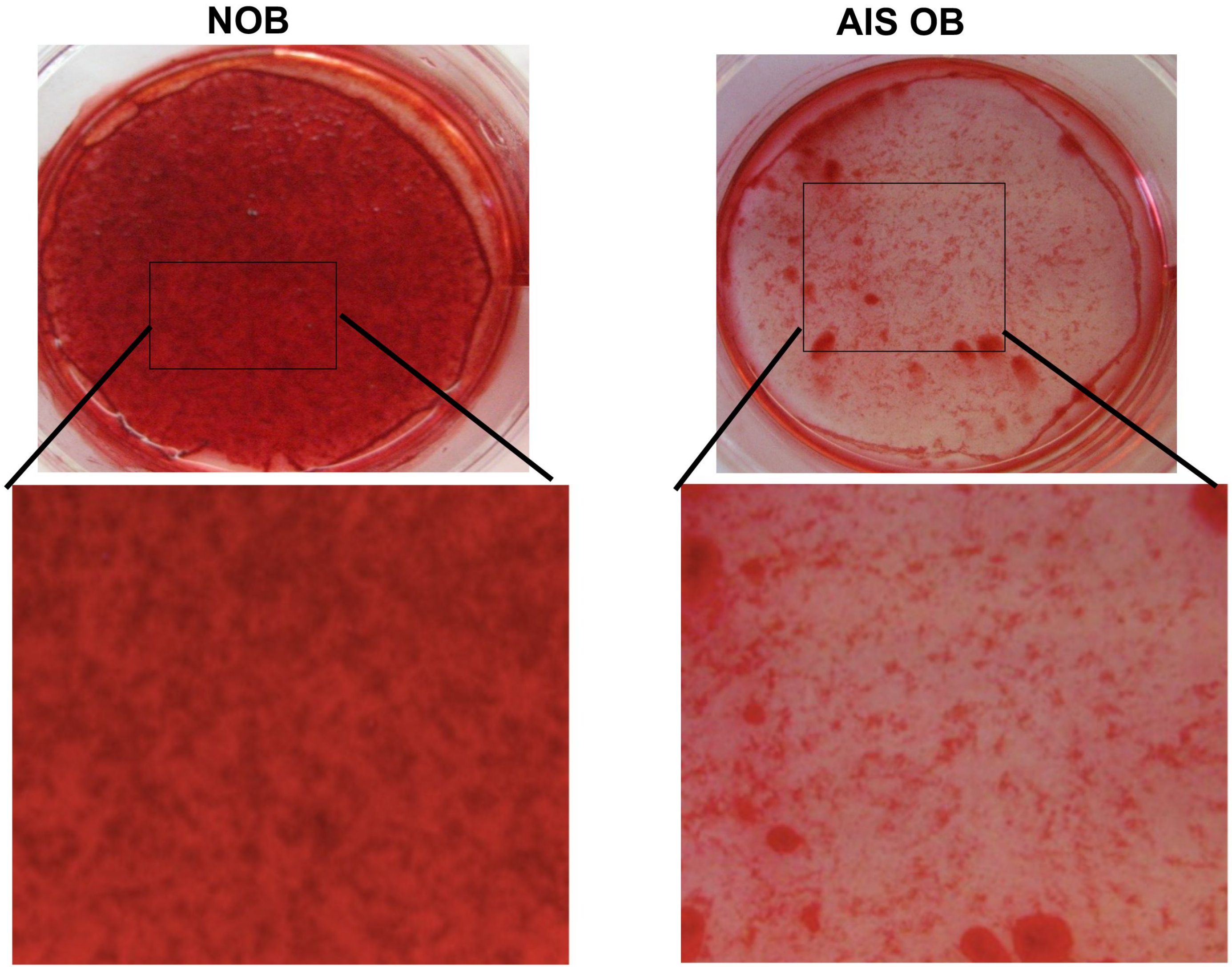

Figure S1 Alizarin red staining. Microscopic view of osteoblasts (OB) from normal (NOB) and AIS patients stained with alizarin red. There is reduced mineral deposition in the AIS cells compared to NOB cells.

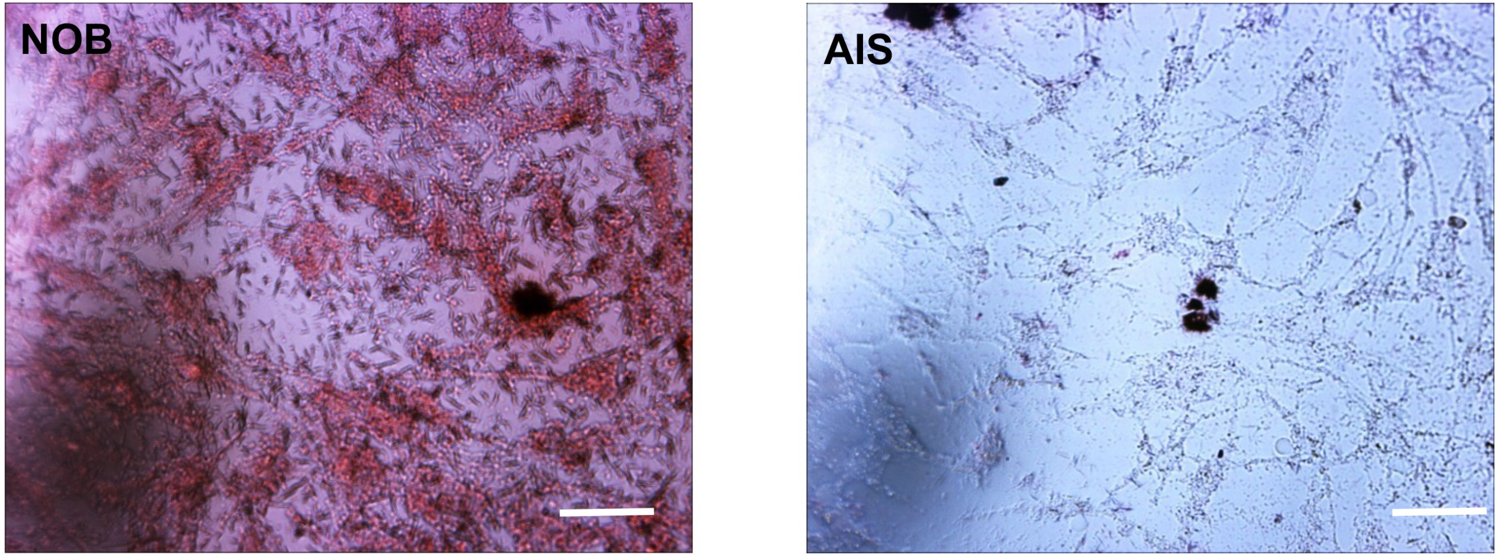

Figure S2 Alkaline phosphatase staining of NOB and AIS cells. Cells were fixed and stained to detect ALP as described in materials and methods. There is stronger staining in NOB cells than in AIS cells. Mag x20, Scale bar 20μm

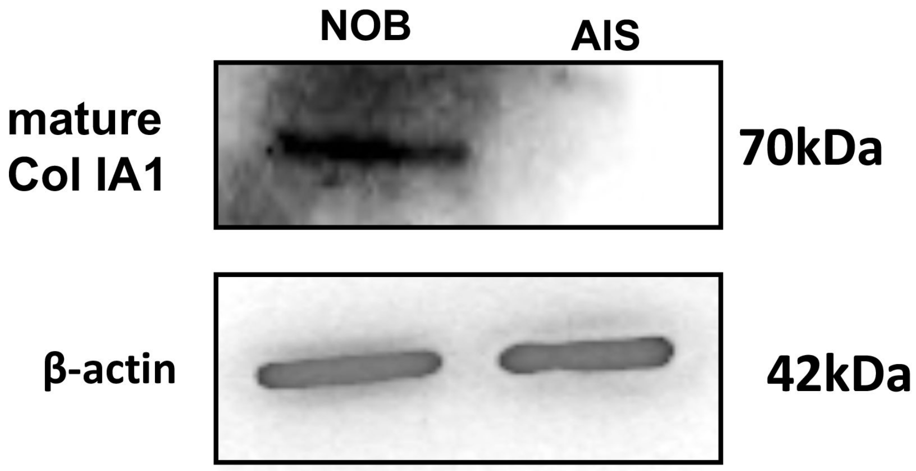

Figure S3 Collagen1A1 protein expression in NOB and AIS cells. AIS cells have lower expression levels of the mature forms of Col1A1

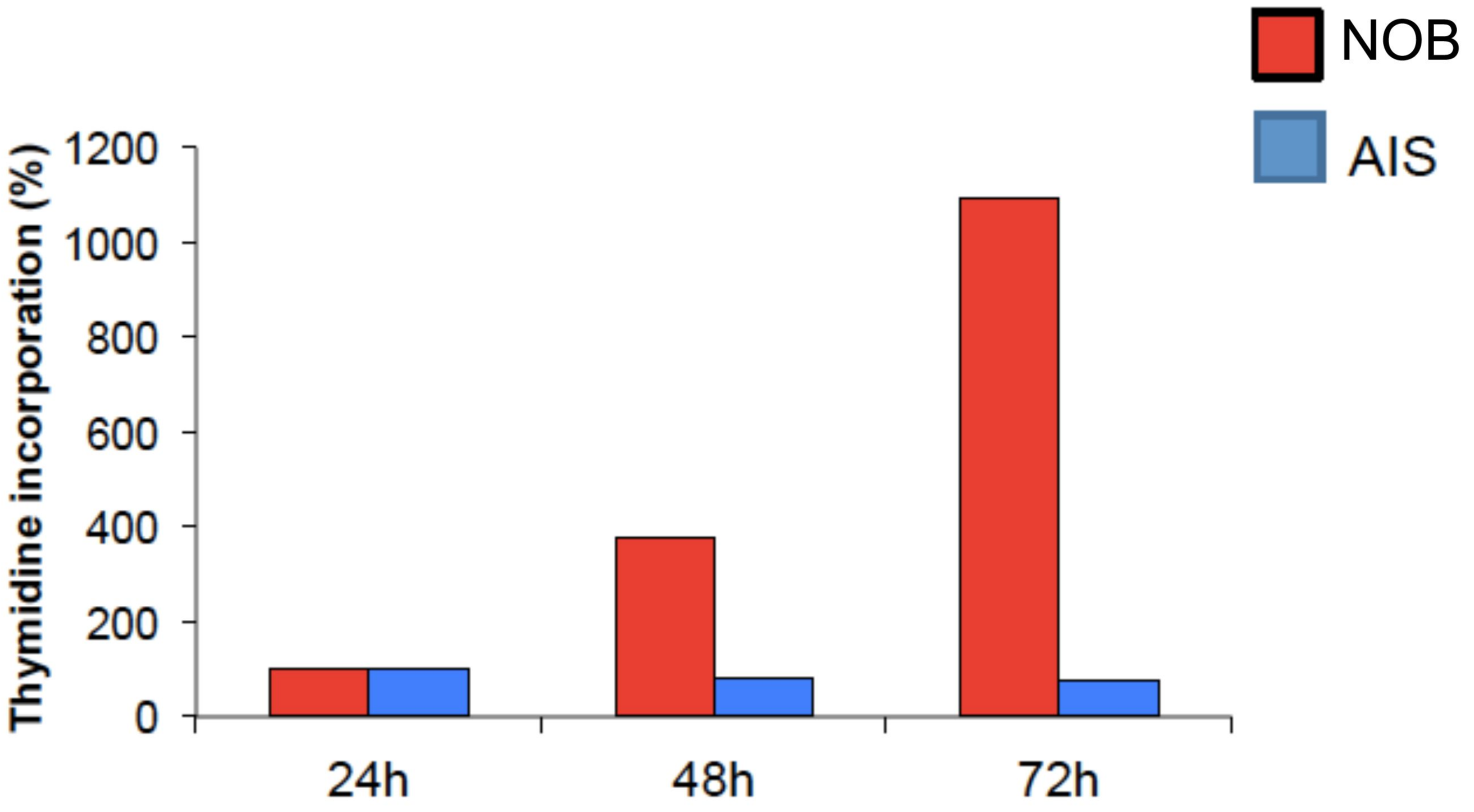

Figure S4 ATP incorporation assay. Proliferation of NOB and AIS cells was determined at different time points (24h, 48h and 72h). AIS cells have delayed proliferation rate compared to NOB cells.
